# The 50 Highest Cited Papers on Shoulder Arthroplasty

**DOI:** 10.3390/healthcare10102000

**Published:** 2022-10-11

**Authors:** Michele Mercurio, Erminia Cofano, Filippo Familiari, Katia Corona, Simone Cerciello, Giorgio Gasparini, Olimpio Galasso

**Affiliations:** 1 Department of Orthopaedic and Trauma Surgery, “Mater Domini” University Hospital, V.le Europa (loc. Germaneto), “Magna Græcia” University, 88100 Catanzaro, Italy; 2 Department of Medicine and Health Sciences “Vincenzo Tiberio”, University of Molise, Via Giovanni Paolo II, 86100 Campobasso, Italy; 3 Casa di Cura Villa Betania, 00165 Rome, Italy; 4 Department of Orthopaedic and Trauma Surgery, Marrelli Hospital, 88900 Crotone, Italy; 5 A. Gemelli University Hospital Foundation IRCCS, Catholic University, 00168 Rome, Italy

**Keywords:** shoulder arthroplasty, rotator cuff, humerus fracture, glenohumeral arthritis, shoulder biomechanics, reverse total shoulder arthroplasty

## Abstract

The purpose of this study was to determine the 50 most cited articles on shoulder arthroplasty (SA) and their characteristics. The Thomson ISIWeb of Science was searched with the following search terms: “shoulder arthroplasty”, “shoulder replacement”, “shoulder prosthesis” and “shoulder implant”. All papers dealing with SA, including its perioperative and postoperative management, were included in this study. Citations ranged from 797 to 52 for the 50 highest cited papers on SA. According to absolute numbers, the top 10 papers were cited at least 118 times. Overall, 78% (n = 43) were clinical and the remaining articles were basic science research (one anatomic, six biomechanical). The most prevalent level of evidence was IV (72%). The *Journal of Shoulder and Elbow Surgery* published 40% of the studies. The majority of studies were conducted in the United States and eight other countries. The publication years of the most-cited articles ranged from 1991 to 2020, with the 2000s accounting for the most articles (96%) and the period from 2006 to 2010 with the absolute largest number of articles (17). This article provides a building block in the SA surgery.

## 1. Introduction

Shoulder arthroplasty (SA) represents one of the most significant technological advancements in shoulder reconstructive surgery and has become extremely popular for patients with degenerative and traumatic injuries to restore shoulder functionality and relive pain. The most common indications for SA are osteoarthritis, inflammatory arthritis, proximal humerus fractures, irreparable rotator cuff tears, rotator cuff arthropathy, and avascular necrosis of the humeral head [1].

SA is now more than 120 years old; the pioneer of this type of surgery was Jules Emile Péan, who implanted the first prosthetic shoulder arthroplasty made of platinum and rubber for the treatment of a patient with shoulder tuberculosis arthritis in 1893. After many design iterations, the Neer’s and the Grammont’s concepts have become the two gold standards. Indeed, the use of arthroplasty was limited for the treatment of shoulder problems until 1955, when Charles Neer reported the use of shoulder prosthesis for persistent pain following humeral head resection for fractures of the neck of the humerus with dislocation of the head fragment [2]. The reverse total shoulder arthroplasty (RTSA) was also introduced in 1974 by Neer and has considerably progressed ever since. The great novelty of RTSA was its ability to also treat rotator cuff deficiency. Nevertheless, Neer’s first RTSA design had several limitations, such as glenoid component loosening and implant breakage because of fairly constrained designs as well as a lateralization of the center of rotation. In 1985, Paul Grammont introduced the novel “ball-and-socket” design, medializing the center of rotation in a non-anatomic location relying on deltoid function alone, and placing lesser demand on the glenoid.

Over the past decade, worldwide implantation rates of SA have drastically increased [3]. In Italy, during 2020 1246 SA implants were performed; over 83% of these were RTSAs. Overall, the diagnosis was osteoarthritis and fracture in 50% and 33% of cases, respectively [4]. The 18th National Joint Registry reported 3833 SA implants in 2021, of which 60% were RTSAs [5].

Analyses of most-cited papers have been performed in the orthopedic field and in a variety of orthopedic subspecialties, including arthroscopy, trauma, anterior cruciate ligament, and hand arthroplasty [6,7,8,9,10]. Holzer et al. [11] focused on arthroplasty surgery and established a ranking of the 50 most-cited papers in hip and knee arthroplasty. However, no such study has been done focusing on SA. The purpose of this study was to determine the 50 most-cited articles on SA and their characteristics.

## 2. Materials and Methods

### 2.1. Search Strategy

In February 2022, the Thomson ISIWeb of Science was searched with the following search terms: “shoulder arthroplasty”, “shoulder replacement”, “shoulder prosthesis” and “shoulder implant”. 

### 2.2. Inclusion Criteria

Articles published in all years and all orthopedic journals were included. Articles were sorted by the number of times cited, from highest to lowest. Each article was evaluated independently by 2 authors (M.M. and E.C.) to determine whether it was appropriately related to SA, first checking the title and/or abstract and then, if necessary, reading the full text. Any discrepancy between authors was resolved by consensus [12]. This process was repeated until 50 articles on SA were retrieved.

Following the methods of Lefaivre et al. [8] and Namdari et al. [13], each article in the 50 most-cited articles was fully analyzed independently by 2 authors (M.M. and E.C.) and the following information was recorded: authors, journal of publication, year of publication, number of citations, geographic origin of corresponding author, and article type (basic science article or clinical research article). All papers dealing with SA, including its perioperative and postoperative management were included in this study. For basic science articles, articles were subtyped as 1 of the following: (1) biomechanical or (2) anatomical. For clinical articles, articles were subtyped as 1 of the following: (1) case series, (2) cohort study, (3) review article, (4) case-control study. Any discrepancy between authors was resolved by consensus. For each clinical article, the level of evidence was determined independently by 2 authors (M.M. and E.C.) based on guidelines published by *The Journal of Bone and Joint Surgery*, American volume [14]. Any discrepancy between authors was resolved by consensus. Following the method of Lefaivre et al. [8], each article was also classified as methodological (i.e., introducing or testing a classification/scoring system or surgical technique) or nonmethodological.

## 3. Results

Citations ranged from 797 to 52 for the 50 most-cited papers on SA [13,15,16,17,18,19,20,21,22,23,24,25,26,27,28,29,30,31,32,33,34,35,36,37,38,39,40,41,42,43,44,45,46,47,48,49,50,51,52,53,54,55,56,57,58,59,60,61,62,63]. The top 10 papers according to absolute numbers were cited at least 118 times [26,27,31,32,39,40,45,50,53,57]. The 50 most-cited papers according to the number of citations are reported in Table 1. 

The distribution of all subtypes is reported in Table 2. 

Most papers (40%) were published in the *Journal of Shoulder and Elbow Surgery*. Other journals include the *Journal of Bone and Joint Surgery* (British Volume), *Journal of Bone and Joint Surgery* (American Volume), *Orthopaedics and Traumatology: Surgery and Research*, *Acta Orthopaedica*, *Journal of Orthopaedics Research: official publication of the orthopaedic research society*, *Clinical Orthopaedics and Related Research*, and *International Orthopaedics*. The distribution of the most-cited papers in the various journals is reported in Table 3. 

Articles originated from 9 countries, with the United States of America having the most contributions (n = 24), followed by France (n = 8), England (n = 6), Switzerland (n = 4), Germany (n = 3), Australia (n = 2), and Canada, Belgium and Norway with 1 each. The publication years of the most-cited articles ranged from 1991 to 2020, and the period from 2006 to 2010 had the absolute largest number of articles (17) (Table 4). 

O. Levy had the most first-author publications, with lead authorship in 3 articles in the top 50 list [39,40,41], and several authors (M. A. Wirth [58,59], S. Gutierrez [32,33], A. Young [62,63]) had 2 lead authorships in the top 50.

## 4. Discussion

In this study, the Thomson ISI Web of Science was searched to analyze the highest cited papers in SA and to define a list of “citation classics” in this subspecialty. The previous publication trends may reflect that in addition to the incremental development of the SA field, in more recent times there have been some major advances or paradigm shifts. It is not necessary that highly cited studies are also the ones that bring about the greatest change in practice [64]. Indeed, a high number of total citations may not be the only parameter to determine the influence or clinical importance. This list of the top 50 most-cited articles in SA provides seminal papers in this field for historical, research, and educational purposes, identifying authors and topics that have had profound influence on the progression of the field. The articles within this list can help elucidate the evolution of standard practice and controversies as a guide for future research and clinical practice. They may also be used in the development of reference lists for resident and fellowship training. 

By analyzing the characteristics of these landmark articles, we sought to determine the characteristics that make these works important. The number of times an article is cited is dependent on several factors. These include, but are not limited to, the quality of the work, the overall interest in the topic discussed, and the ability of the article to inspire further research or to change the way in which physicians practice or conduct research. An additional factor in the evolution of literature is the maturity, or conversely, the novelty of a subspecialty. Articles written during the development or inception of a field will provide building blocks for the advancement within the field and will likely be highly cited. However, it is important to note that we did not evaluate included studies for their quality other than for their level of evidence; hence, the articles included in the top 50 list do not necessarily represent the best clinical or basic science, as evidenced by the many level IV studies included in the list. Curricula that include this list of articles should therefore continue to be implemented with textbooks, instructional course lectures, more recently published articles, and other sources that are deemed educationally important.

The majority of articles included originated in the United States, which also has been found in reviews of other specialties such as anesthesia [65,66], plastic surgery [67], general surgery [68], and orthopedic surgery in general [69]. In total, nine countries contributed to the top 50 list of most-cited papers in SA arthroplasty. All countries are highly industrialized and are ranked among the top in both economical and health-care expenditure. As noted by Holzer et al. [11], this reflects the high level of orthopedic surgery in these countries, especially in a field with highly advanced technical methods and highly sophisticated implants.

It is also not surprising that most of the articles in the top 50 have been published in the past two decades, because the SA procedure is newer than hip and knee arthroplasties and has been performed and analyzed more frequently since the 2000s. In this context, it should be noted that the most-cited paper on hip arthroplasty was cited 2495 times [11], while the most-cited paper on SA reached 797 citations. Hip arthroplasty was introduced first and served as a model for arthroplasties in other joints [70]. Furthermore, these data underline the magnitude of hip arthroplasties: epidemiological data from the past decades show that the highest number of arthroplasties has been performed in the hip joint [71].

The first three articles present in the list, written by Sirveaux et al. [50], Werner et al. [57], and Frankle et al. [26], discussed RTSA as more than half of the articles in this list; the first three articles also were written in 2004 and 2005. This showed the great interest in this type of prosthesis. Indeed, the indications for RTSA expanded over time and several shoulder pathologies other than massive, irreparable rotator cuff tear can be successfully treated with RTSA (i.e., fractures, glenohumeral arthritis, tumors of the proximal humerus, revision of failed hemiarthroplasty or total shoulder, etc.). In the 50 most-cited papers on SA, several articles focused on biomechanics such as the options for the fixation of the glenoid component or the muscle angle force. The study of biomechanics is very important in RTSA for the preservation of soft-tissue balancing, the postoperative range of motion, the shoulder functionality, and the long-term survivorship of the implant.

### Limitations

This study has a number of weaknesses. First, 50 articles, as within any other number, is arbitrary, and there are important and influential articles that were not included; however, we believe that 50 represents a reasonable number of articles to take into consideration as building blocks. Second, the total number of citations is inherently susceptible to sources of bias. We did not exclude author self-citations; authors may be more likely to cite articles in journals in which they seek publication, and this is a source of bias that we were unable to control. We did not include citations in the so-called gray literature (textbooks, lectures, or other non-peer-reviewed and web-based literature). A final weakness is that, as theorized by Kuhn, authors are more likely to cite articles because of previous citations, rather than independently analyzing them for content and quality [72]. Because the goal of our study was to use the total number of citations as a means of creating a list of highly influential SA articles, we do not believe that these limitations significantly influence our conclusions. As stated by Eom et al. [64], the current work helps young researchers who, however, should not prioritize the citation number exclusively over the content of the article as it is not necessary that highly cited studies are also the ones that bring about the greatest change in practice.

## 5. Conclusions

This article provides clinicians, researchers, and trainees with a group of articles that should be taken into consideration as building blocks in shoulder arthroplasty surgery. Furthermore, the selection of these articles is useful for learning more about current trends in shoulder arthroplasty and anticipating future developments.

## Figures and Tables

**Table 1:** Top 50 cited shoulder arthroplasty papers.

Rank	Author(s)	Title	No. of Citations	Year	Country	Article Type	Type of Study (loe)
1	Sirveaux et al.	Grammont inverted total shoulder arthroplasty in the treatment of glenohumeral osteoarthritis with massive rupture of the cuff—Results of a multicentre study of 80 shoulders	797	2004	France	CR (cohort study)	IV
2	Werner et al.	Treatment of painful pseudoparesis due to irreparable rotator cuff dysfunction with the delta III reverse-ball-and-socket total shoulder prosthesis	598	2005	Switzerland	CR (cohort study)	IV
3	Frankle et al.	The reverse shoulder prosthesis for glenohumeral arthritis associated with severe rotator cuff deficiency—A minimum two-year follow-up study of sixty patients	524	2005	USA	CR (case series)	IV
4	Torchia et al.	Total shoulder arthroplasty with the Neer prosthesis: Long-term results	435	1997	USA	CR (case series)	IV
5	Guery et al.	Reverse total shoulder arthroplasty—Survivorship analysis of eighty replacements followed for five to ten years	428	2006	France	CR (cohort study)	IV
6	Gallinet et al.	Three or four parts complex proximal humerus fractures: Hemiarthroplasty versus reverse prosthesis: A comparative study of 40 cases	194	2008	France	CR (cohort study)	IV
7	Gutièrrez et al.	Range of Impingement-Free Abduction and Adduction Deficit After Reverse Shoulder Arthroplasty Hierarchy of Surgical and Implant-Design-Related Factors	168	2008	USA	BS (Biomechanics-computer model)	V
8	Levy et al.	Cementless surface replacement arthroplasty of the shoulder—5-to 10-year results with the Copeland mark-2 prosthesis	158	2001	England	CR (cohort study)	IV
9	Levy et al.	Cementless surface replacement arthroplasty (Copeland CSRA) for osteoarthritis of the shoulder	121	2004	England	CR (case series)	IV
10	Melis et al.	An evaluation of the radiological changes around the Grammont reverse geometry shoulder arthroplasty after eight to 12 years	118	2011	France	CR (cohort study)	IV
11	Gutierrez et al.	Biomechanical comparison of component position and hardware failure in the reverse shoulder prosthesis	101	2007	USA	BS (Biomechanics)	V
12	Martin et al.	Uncemented glenoid component in total shoulder arthroplasty—Survivorship and outcomes	100	2005	USA	CR (cohort study)	IV
13	Boileau et al.	Revision surgery of reverse shoulder arthroplasty	100	2013	France	CR (Case Series)	IV
14	Hopkins et al.	The effects of glenoid component alignment variations on cement mantle stresses in total shoulder arthroplasty	96	2004	England	BS (biomechanics studies	II
15	Wright et al.	Humeral fractures after shoulder arthroplasty	95	1995	USA	CR (case series)	IV
16	Kumar et al.	Periprosthetic Humeral Fractures After Shoulder Arthroplasty	94	2004	USA	CR (case series)	IV
17	Fevang et al.	Risk factors for revision after shoulder arthroplasty 1825 shoulder arthroplasties from the Norwegian Arthroplasty Register	93	2009	Norway	CR (case series)	IV
18	Gilles Walch et al.	Do the indications, results, and complications of reverse shoulder arthroplasty change with surgeon’s experience?	86	2012	France	CR (Retrospective Case Control)	III
19	Levy et al.	Copeland surface replacement arthroplasty of the shoulder in rheumatoid arthritis	85	2004	England	CR (case series)	IV
20	Bailie et al.	Cementless Humeral Resurfacing Arthroplasty in Active Patients Less Than Fifty-five Years of Age	84	2008	USA	CR (cohort study)	IV
21	Young et al.	A multicentre study of the long-term results of using a flat-back polyethylene glenoid component in shoulder replacement for primary osteoarthritis	82	2011	Australia	CR (cohort study)	IV
22	Huget et al.	Results of a new stemless shoulder prosthesis: Radiologic proof of maintained fixation and stability after a minimum of three years’ follow-up	82	2010	France	CR (case series)	IV
23	Young et al.	Secondary Rotator Cuff Dysfunction Following Total Shoulder Arthroplasty for Primary Glenohumeral Osteoarthritis: Results of a Multicenter Study with More Than Five Years of Follow-up	82	2012	Australia	CR (cohort study)	IV
24	Yian et al.	Radiographic and Computed Tomography Analysis of Cemented Pegged Polyethylene Glenoid Components in Total Shoulder Replacement	81	2005	Switzerland	CR (cohort study)	IV
25	Trail et al.	The results of shoulder arthroplasty in patients with rheumatoid arthritis	81	2002	England	CR (cohort study)	IV
26	Jobin et al.	Reverse total shoulder arthroplasty for cuff tear arthropathy: the clinical effect of deltoid lengthening and center of rotation medialization	80	2012	USA	CR (prospective cohort study)	II
27	Cil et al.	Survivorship of the humeral component in shoulde arthroplasty	73	2010	USA	CR (case series)	IV
28	Athwal et al.	Periprosthetic Humeral Fractures During Shoulder Arthroplasty	73	2009	USA	CR (cohort study)	IV
29	Berliner et al.	Biomechanics of reverse total shoulder arthroplasty	72	2014	USA	Narrative rewiev	IV
30	Nelson et al.	Outcomes in the treatment of periprosthetic joint infection after shoulder arthroplasty: a systematic review	71	2016	USA	Systematic review	IV
31	Wirth et al.	Treatment of Glenohumeral Arthritis with a Hemiarthroplasty: A Minimum Five-Year Follow-up Outcome Study	71	2006	USA	CR (cohort study)	IV
32	Wirth et al.	Total Shoulder Arthroplasty with an All-Polyethylene Pegged Bone-Ingrowth Glenoid Component A Clinical and Radiographic Outcome Study	67	2012	USA	CR (cohort study)	IV
33	Thomas et al.	Outcome of Copeland surface replacement shoulder arthroplasty	66	2005	England	CR (case series)	IV
34	De Wilde et al.	Shoulder prostheses treating cuff tear arthropathy: a comparative biomechanical study	65	2004	Belgium	BS (Biomechanics)	II
35	Orfaly et al.	A prospective functional outcome study of shoulder arthroplasty for osteoarthritis with an intact rotator cuff	64	2003	USA	CR (cohort study)	III
36	Namdari et al.	Comparison of Hemiarthroplasty and Reverse Arthroplasty for Treatment of Proximal Humeral Fractures A Systematic Review	64	2013	USA	Systematic review	IV
37	Favre et al.	The effect of component positioning on intrinsic stability of the reverse shoulder arthroplasty	62	2010	Switzerland	BS (Biomechanics)	II
38	Kasten et al.	Mid-term survivorship analysis of a shoulder replacement with a keeled glenoid and a modern cementing technique	61	2009	Germany	CR (cohort study)	IV
39	Mollon et al.	Impact of scapular notching on clinical outcomes after reverse total shoulder arthroplasty: an analysis of 476 shoulders	61	2017	USA	CR (Retrospective cohort study)	III
40	Gallo et al.	Instability after reverse total shoulder replacement	59	2011	USA	CR (case series)	IV
41	Matsen et al.	Factors Affecting Length of Stay, Readmission, and Revision After Shoulder Arthroplasty A Population-Based Study	57	2015	USA	CR (cohort study)	III
42	Giles et a.	Implant Design Variations in Reverse Total Shoulder Arthroplasty Influence the Required Deltoid Force and Resultant Joint Load	56	2015	Canada	BS (cadaver)	IV
43	Wagner et al.	Glenoid Bone-Grafting in Revision to a Reverse Total Shoulder Arthroplasty	55	2015	USA	CR (cohort study)	III
44	Loew et al.	Influence of the design of the prosthesis on the outcome after hemiarthroplasty of the shoulder in displaced fractures of the head of the humerus	55	2006	Germany	CR (cohort study)	IV
45	Cazeneuve et al.	Long term functional outcome following reverse shoulder arthroplasty in the elderly	54	2011	France	CR (cohort study)	IV
46	Throckmorton et al.	Pegged versus keeled glenoid components in total shoulder arthroplasty	54	2010	USA	CR (case control study)	III
47	Gerber et al.	Longitudinal observational study of reverse total shoulder arthroplasty for irreparable rotator cuff dysfunction: results after 15 years	53	2017	Switzerland	CR (case series)	IV
48	Nam et al.	Observations on retrieved humeral polyethylene components from reverse total shoulder arthroplasty	53	2010	USA	BS (analysis of component)	II
49	Aldinger et al.	Complications in shoulder arthroplasty: an analysis of 485 cases	52	2009	Germany	CR (case series)	IV
50	Codsi et al.	The effect of screw position on the initial fixation of a reverse total shoulder prosthesis in a glenoid with a cavitary bone defect	52	2008	USA	CR (cohort study)	IV

BS, basic science; CR, clinical research; LOE, level of evidence.

**Table 2:** Subtypes of the 50 most-cited papers on shoulder arthroplasty.

Subtype	No. of Articles
**Clinical research**	
Cohort study	24
Case series	14
Review article	3
Case control	2
**Basic science**	
Biomechanical study	6
Anatomical study—cadaver	1

**Table 3:** Number of citations on top 50 list by source journal.

Journal	No. of Articles
Journal of Shoulder and Elbow Surgery	20
Journal of Bone and Joint Surgery (American Volume)	17
Journal of Bone and Joint Surgery (British Volume)	7
Orthopaedics and Traumatology: Surgery and Research	2
Acta orthopaedica	1
Journal of Orthopaedics Research: official publication of the orthopaedic research society	1
Clinical Orthopaedics and Related Research	1
International Orthopaedics	1

**Table 4:** Years of publication of the 50 most-cited papers on shoulder arthroplasty.

Year of Publication	No. of Articles
1991–1995	1
1996–2000	1
2001–2005	14
2006–2010	17
2011–2015	14
2016–2020	3

## Data Availability

Not applicable.
